# Self-Healing of Pluronic® F127 Hydrogels in the Presence of Various Polysaccharides

**DOI:** 10.3390/gels9090719

**Published:** 2023-09-05

**Authors:** Alexandra Lupu, Luiza Madalina Gradinaru, Daniela Rusu, Maria Bercea

**Affiliations:** “Petru Poni” Institute of Macromolecular Chemistry, 41-A Grigore Ghica Voda Alley, 700487 Iasi, Romania; gradinaru.luiza@icmpp.ro (L.M.G.); rusu.daniela@icmpp.ro (D.R.)

**Keywords:** Pluronic® F127, thermoresponsive hydrogels, xanthan gum, shear-thinning, yield stress, self-healing ability

## Abstract

Thermoresponsive Pluronic® F127 (PL) gels in water were investigated through rheological tests in different shear conditions. The gel strength was tuned with the addition of 1% polysaccharide solution. In the presence of xanthan gum (XG), the viscoelastic behavior of PL-based hydrogels was improved in aqueous environment, but the rheological behavior was less changed with the addition of XG in PBS solutions, whereas in the presence of 0.1 M NaCl, the viscoelastic parameters decreased. PL micellar networks exhibited a self-healing ability, recovering their initial structure after applying cycles of high strain. The rheological characteristics of the PL hydrogel changed with the addition of 1% polysaccharides (xanthan gum, alginate, κ-carrageenan, gellan, or chitosan). PL/polysaccharide systems form temperature-responsive hydrogels with shear thinning behavior, yield stress, and self-healing ability, being considered a versatile platform for injectable biomaterials or bioinks. Thus, in the presence of xanthan gum in aqueous medium, the gel strength was improved after applying a high strain (the values of elastic modulus increased). The other investigated natural polymers induced specific self-healing behaviors. Good performances were observed with the addition of gellan gum, alginate, and κ-carrageenan, but for high values of strain, the ability to recover the initial structure decreased. A modest self-healing behavior was observed in the presence of chitosan and xanthan gum dissolved in NaCl solution.

## 1. Introduction

**Scheme 1:**
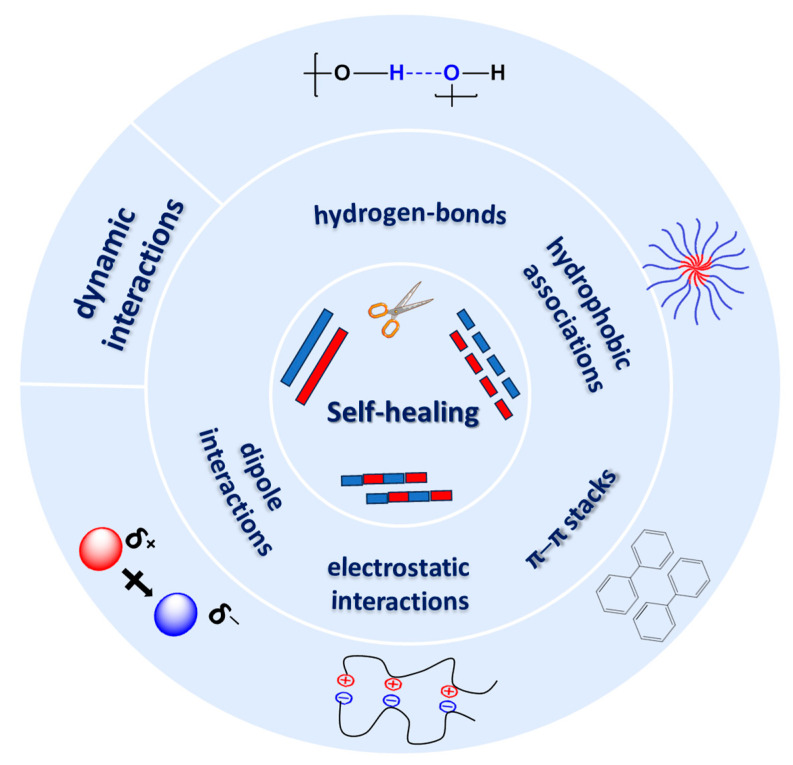
Schematic presentation of various types of interactions responsible for the self-healing behavior of hydrogels.

Hydrogels based on biomolecules are widely used materials in the biomedical field, due to their biocompatibility, low toxicity, high permeability to metabolites or nutrients, and water swelling ability. Among all polysaccharides, alginate, chitosan, hyaluronic acid, cellulose, dextran, and their derivatives have been intensively studied as hydrogel components during the last decades [1]. Polysaccharides have a low immunogenic profile, are easily handled, and have relatively low costs, aspects which make them preferred candidates for hydrogel fabrication [2]. One of the most important advantages of polysaccharide-based hydrogels is their self-healing ability, which implies that these materials have the capacity to self-repair and autonomously recover their original structure after damage [3]. The self-healing mechanisms require particular functionalities, in order to provide linkages between the injured parts of the gel. These mechanisms are based on dynamic interactions, which are either dynamic covalent crosslinking or physical interactions (supramolecular assembly) (Scheme 1), such as hydrophobic associations, ionic crosslinking, or hydrogen bonding networks [4]. Thus, self-healing hydrogels have attracted attention for tissue engineering applications, such as skin regeneration enhancement, due to their extracellular matrix (ECM)-like damage-healing properties [2].

In order to ensure their bio-applicability, hydrogels require stimuli-responsive properties, such as temperature-, light-, pH-, electric-, or magnetic field-responsive abilities. Polymers that exhibit thermoresponsive behavior present a low critical solution temperature (LCST), being able to reversibly change upon heating from a hydrophilic state (non-associative) to a hydrophobic state (associative) [5]. At low temperature (below LCST), aqueous solutions of temperature-responsive polymers are in a sol state (characterized by viscous flow), which allows them to be easily injected into a higher temperature environment, above the LCST (e.g., body temperature). Thus, due to the association of polymer chains, these gelators form a physical network (gel state with solid-like behavior) at the injection site [5].

Pluronics play an important role as polymeric excipients and they have been extensively used as stabilizers, solubility improvers, bioavailability enhancers, and thermosensitive drug carriers [6]. Pluronic® F127 (PL) is an FDA-approved, non-toxic and water-soluble triblock copolymer made of two hydrophilic poly(ethylene oxide) blocks and one hydrophobic poly(propylene oxide) block placed in the middle of the hydrophilic segments (PEO_100_–PPO_65_–PEO_100_). PL is one of the most commonly used thermoresponsive polymers and exhibits a rapid sol–gel transition above the critical gelation temperature and concentration [7], when the hydrophilic PEO blocks stretch out, whereas the hydrophobic PPO sections start to associate and form a dense core, resulting a network structure [8]. Thus, due to their temperature-sensitive characteristics that allow effortless injectability, PL-based hydrogels are widely used in tissue engineering and drug delivery applications [9,10,11]. However, in contact with an excess of water, PL gels disintegrate within several days [12].

In this context, many efforts have been carried out to improve the gel performances, stability over time, viscoelastic and biological properties of PL gels [6,12,13,14,15,16]. Polysaccharide addition appears to be a convenient way to ensure the shape stability, gel stabilization against dissolution [12,17] and biocompatibility [18] of PL-based systems. The gels are stabilized by the interactions of long polysaccharide chains with PEO blocks, which can be combined with the hydrophobic interactions of hydrocarbon chains (>C16) with the core of the Pluronic micelles [12]. The PL micelles surrounded by the polysaccharide chains cannot be separated from the micelles network, and thus the composite gel is more stable in an excess of solvent (water or biological fluid) [12]. However, the stability of Pluronic gels slowed down in the presence of nonionic polysaccharide, and pullulan addition in concentrated systems caused a phase separation [18].

In recent years, PL-polysaccharides-based hydrogels have been discussed in many studies, especially from a biomedical and pharmaceutical point of view [18,19,20]. These materials are popular for drug-delivery systems, biosensor formulations, and tissue engineering applications. Cellulose, hyaluronic acid, chitosan, alginate, starch, and gellan gum are some of the most used polysaccharides in the preparation of hydrogels with injectable and self-healing properties. The functional groups of these polysaccharides, such as amino (–NH_2_), carboxyl (–COOH), aldehyde (–CHO), and hydroxyl (–OH), are involved in hydrophobic interactions, which allow them to form physical networks [12,13,21,22,23,24].

The (cyto)toxicity of PL was deeply investigated, in order to evaluate the biocompatible properties and nontoxicity of the pure copolymer compared to other Pluronics (e.g., Pluronic P94) [25]. A series of studies dealt with the increase in the cytotoxicity of PL gels with chemical functionalization with different compounds [26,27]. For example, α-tocopherol (TOC) was used as a toxicity enhancer grafted on PL. As the study concluded, PL–SS–TOC showed low cytotoxicity, with an in vitro cell viability of about 85% after 48 h incubation. Thus, the results demonstrated the potential of PL–SS–TOC in the biomedical area [26]. Li et al. [27] reported the synthesis of a novel folated PL modified liposome (cur–FA–PL–Lps) by attaching folic acid (FA) to PL chains via a dehydration condensation reaction, followed by curcumin incorporation. Cytotoxicity studies of cur–FA–PL–Lps demonstrated a cell viability of about 90–100% after 24 h of incubation, which indicates that the complex was not toxic to cells.

The present paper first focused on the rheological behavior of PL hydrogels in the presence of xanthan gum (XG). Then, the self-healing behavior of PL/XG gels was compared with those of PL-based gels that contain other polysaccharides: alginate (Alg), κ-carrageenan (κ-Carr), gellan gum (GG), and chitosan (CS). The main interest was to obtain homogeneous and low-viscosity formulations at ambient temperature that undergo a sol–gel transition under physiological conditions, developing shear thinning and elastic networks with self-healing ability. How the addition of different polysaccharides changed the gel behavior after undergoing high deformations was investigated, as encountered in many applications. Pluronic® F127 was selected due to its wide-spread use in pharmaceutical formulations and its FDA approval for intravenous use in humans [25]. The Pluronics with higher PPO content exhibited cytotoxicity, due to their ability to incorporate in the cellular membrane, altering its viscosity, and also reducing the intracellular adenozin trifosfat concentration [25].

## 2. Results and Discussion

Table 1 presents the composition of the samples investigated in this paper. We used two PL samples in water, sample 1 of 20% (wt.) and sample 2 of 16.83% (wt.), to compare their behavior with PL/polysaccharide gels in similar conditions. Table 1 also includes the main rheological characteristics of the samples determined in different shear conditions, at constant temperature of 37 °C.

At low PL concentrations, micelles were isolated and the fluid was isotropic. For a sufficiently high concentration, by increasing the temperature, more spherical micelles were formed, increasing their density and determining their arrangement into a network [28,29].

**Figure 1:**
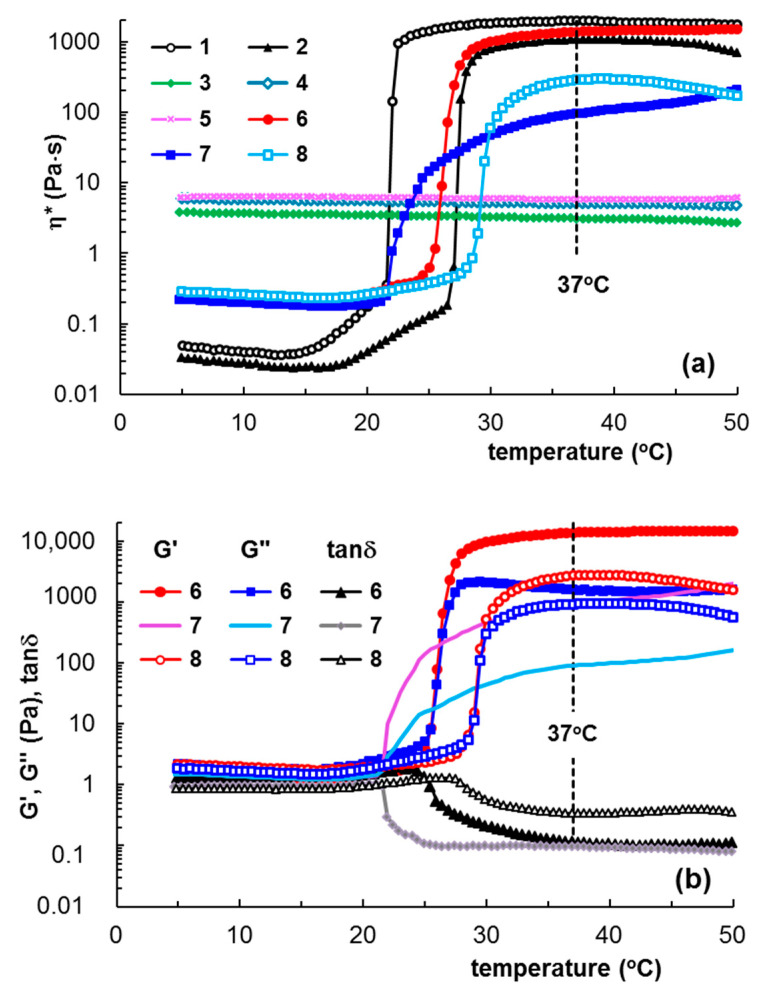
Temperature-induced gelation illustrated through the dependences of (**a**) complex viscosity (η*) for samples 1–8; (**b**) viscoelastic parameters (G′, G″ and tanδ) for PL/XG samples (heating rate of 1 °C/min, ω = 10 rad/s, γ = 1%). All samples were previously stored at 5 °C.

**Scheme 2:**
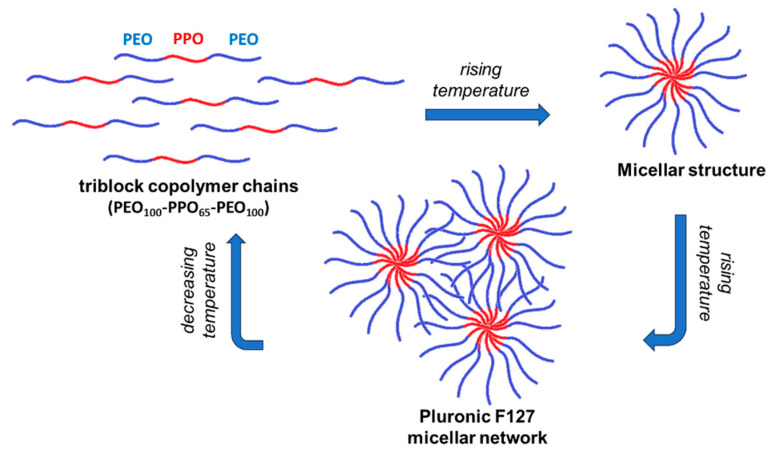
Schematic presentation of temperature-induced gelation for Pluronic® F127.

PL sample of 20% concentration in water is in sol state at low temperature, with a predominantly viscous behavior. It undergoes a sol–gel transition around 21.6 °C and at higher temperatures a solid-like behavior prevails. This transition temperature slowly shifts to higher values as the copolymer concentration decreases; thus, for 16.83% PL, the transition was observed around 27 °C. The temperature-induced gelation of PL (Figure 1) was due to the intensification of hydrophobic interactions, and as the concentration of micelles increased, the degree of hydration of the ethylene oxide units decreased; thus, the hydrogels were rapidly formed by packing of micellar subunits [28,30] through an entropy-driven process [8,28] into a lattice arrangement (lamellar, hexagonal or cubic) [31,32,33]. This thermoreversible micellar mode of chains association (Scheme 2) makes PL attractive for various applications [34,35,36]. Several studies have proposed XG-PL-based hydrogels formulations with potential applications as ophthalmic drug-delivery systems [37,38], buccal drug-delivery systems [39], and for topical application and administration of different active principles [40,41,42]. Injectable Pluronic hydrogels were considered suitable systems to assist local cancer treatment (for chemotherapy, phototherapy, immunotherapy, or gene therapy). One limitation of their use is the high erosion rate of Pluronic hydrogels, which reduces the therapeutic efficacy [43].

The rheological behavior of XG-containing systems is influenced by the anions (from NaCl or PBS solutions) and also by the structure of polysaccharides in an aqueous environment. The effect of XG addition to 20% PL in water was investigated in detail for composite gels by adding aqueous solutions of polysaccharide (samples 6, 9–11), pH = 5, or salted solutions: 0.1 M NaCl solution (sample 7) and phosphate buffered saline (PBS) solution (sample 8), pH = 7.4. Samples with various XG concentrations added to PL gels were preliminary tested using rheology. An optimum concentration of 1% XG was found, with XG addition at this composition able to increase the network strength, as can be observed from the values of the rheological parameters shown in Table 1.

In aqueous solutions, XG macromolecules are able to form high viscous fluids, even at low concentrations, being used as a stabilizer or thickening agent in various applications, including in food, pharmaceutical, cosmetic, agricultural, textile, ceramic, and petroleum industry [44]. The behavior of the XG solutions was not significantly influenced by the temperature increase in the investigated range (up to 50 °C).

### 2.1. Gelation of Pluronic® F127 in the Presence of XG Chains

**Figure 2:**
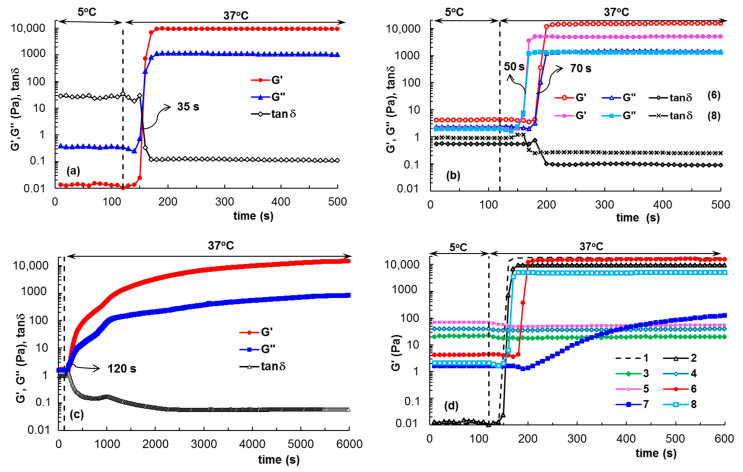
The evolution of viscoelastic parameters during gelation for (**a**) sample 2; (**b**) samples 6 and 8; (**c**) sample 7; (**d**) comparative behavior for samples 1–8. The samples were previously stored at 5 °C, introduced into the rheometer geometry, which was thermostated at 5 °C; firstly, they were monitored at 5 °C for 120 s; then the temperature was suddenly changed to 37 °C and the in situ gelation was followed over time.

The effect of the addition of 1% xanthan gum (XG) in aqueous and salted (0.1 M NaCl and PBS) solutions on the gelation of PL was investigated as a function of temperature and at 37 °C using samples stored at 5 °C, in order to determine the transition temperature (Figure 1) and time (Figure 2). At low temperatures, the PL-based samples behaved as viscous fluids (liquid-like state), with an elastic modulus (G′) lower than the viscous modulus (G″), with the loss tangent having values higher than unity. The sol state of PL was characterized by low values of viscoelastic moduli (G′ and G″), which increased considerably in the presence of XG, when G′ became closer to G″ (tanδ decreased).

**Figure 3:**
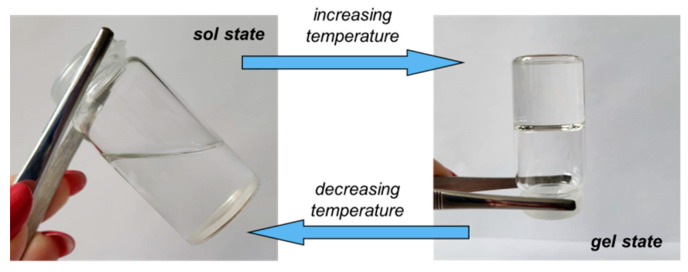
Illustration of the sol and gel states for PL-based hydrogels.

By applying a controlled temperature rise (heating rate of 1 °C/min), the hydrophobic interactions inside the PL-containing samples increased and determined the micelles and polymicelles formation, which further generated a physical network [6,14,15]. Consequently, at the transition point, a jump of the viscoelastic parameters was registered, which allowed the identification of the transition temperature (Figure 1) or the transition time at constant temperature (Figure 2). After the transition point, G′ became higher than G″ and tanδ < 1, suggesting network formation (solid-like behavior). A visual illustration of the sol and gel states is presented in Figure 3.

A well-defined transition point was observed for PL in the aqueous environment, when the sol–gel transition occurred over a narrow temperature domain (around 2 °C), which enlarged slightly in the presence of XG (around 4 °C). For the same polymer concentration, a further increase in temperature above the transition point determined an improvement in the gel strength in the presence of polysaccharide.

In aqueous solutions at low temperatures, the XG macromolecules formed random helix structures through hydrogen bonding [45]. Salt addition reduced the effective charge of XG macromolecule, determining a chain aggregation and the viscosity increases. During heating, the XG chains underwent a disruption of aggregates [46,47], slowing down the dynamics of PL gelation (sample 7, Figure 1a,b and Figure 2c).

**Figure 4:**
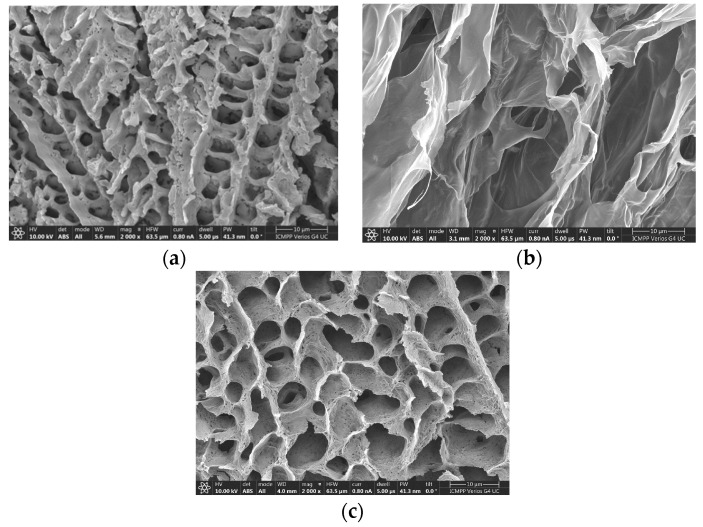
Scanning electron microscopy images of (**a**) sample 2; (**b**) sample 3, and (**c**) sample 5.

Figure 4 shows SEM photographs obtained of the cross-section of samples 2, 3 and 5. The dry PL hydrogel (Figure 4a) exhibited a porous structure with pores of 1–5 μm. In the absence of PL, the XG sample presented large holes, higher than 20–30 μm (Figure 4b).

The pores become interconnected and their size increased to 5–10 μm through adding XG chains into the PL gel (Figure 4c). Similar structures were reported for PL and chitosan derivatives [48]. For the injectable hydrogels used as a scaffold for tissue engineering applications, the pores avoid the passage of nutrients to deliver them to the cells.

Besides temperature and concentration, environmental conditions influence sol–gel behavior. Light scattering studies evidenced that the nature and concentration of salts influences the critical micellization temperature of Pluronics, changing the hydrogen bonding in solution and the water structure [49,50]. In the presence of NaCl, corona-specific dehydration occurs, favoring the formation of micellar clusters [14].

Most pharmaceutical Pluronic-based gels contain phosphate buffers, and it was shown that sodium phosphates have a strong effect on copolymer micellization [49]. In the presence of anions from the buffers used as simulated body fluids, the water–water interactions are enhanced and the entropy increases during micellization. In a phosphate buffer solution, a less organized structure is formed by PL and the aggregates formed during micellization are smaller and more stable, as compared with those observed in pure water [51].

### 2.2. Shear Flow Behavior at 37 °C

**Figure 5:**
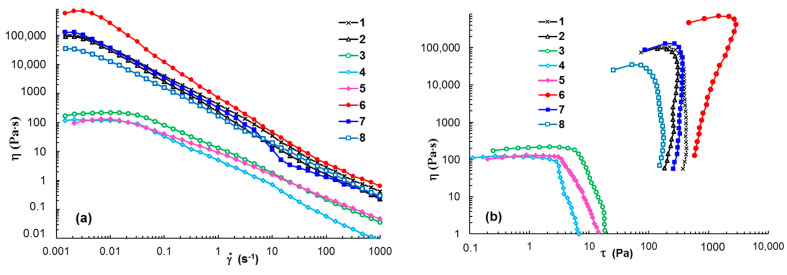
Shear viscosity as a function of (**a**) shear rate and (**b**) shear stress for samples 1–8 (Table 1) at 37 °C.

Figure 5 shows the flow behavior in steady shear conditions for samples 1 to 8, which had previously been thermostated at 37 °C. The PL-based hydrogels exhibited a non-Newtonian behavior over the whole range of investigated shear rates (Figure 5a). In the presence of XG dissolved in water (sample 6), the hydrogel resistance to flow increased and higher values of shear viscosity were obtained. This may have been due to the competition between the hydrodynamic interactions exhibited by different macromolecules in shear conditions [52] or changes in the interactions of PL with water [14].

When XG was dissolved in 0.1 M NaCl (sample 7), a small increase in viscosity was observed for $\dot{\gamma }$ < 10 s^−1^; above this limit, the gel showed flow instabilities and viscosity decreases, suggesting a loss of structural integrity under the action of the intense shearing forces.

The 1% XG in water or salted solutions exhibited Newtonian behavior at low shear rates ($\dot{\gamma }$ < 0.3 s^−1^) and they behaved as non-Newtonian fluids with higher shear rate values, when the viscosity decreased with rising $\dot{\gamma }$.

The yield stress value (σ_o_) is the minimum shear stress applied to a sample for starting the shear thinning flow when the rest of the structure is breaking down. Yield stress analysis is useful for injectable hydrogels [53,54] and bioinks [55,56] when the hydrogels are submitted to high shear forces. From the dependences of shear viscosity as a function of shear stress (Figure 5b), the yield stress values were determined. For the investigated PL-based hydrogels, the yield stress was of the order of a hundred Pa, in agreement with other data in the literature [29]. The high yield stress values of PL-based hydrogels were attributed to repulsive interactions between close-packed spherical micelles [57]. The addition of 1% XG in aqueous and NaCl solutions induced a higher σ_o_ value to the PL hydrogels. Similar effects were observed when adding aqueous solutions of Alg, κ-Carr and GG (Table 1). These systems had sufficient strength to resist tearing once they were stretched (σ_o_ varied from 104.9 Pa in PBS solution to 1080.4 Pa in aqueous medium), and thus they present shape fidelity in 3D printing [58]. The XG in PBS solutions and CS in 1% acetic acid decreased the yield stress value.

### 2.3. Delimitation of Linear and Non-Linear Viscoelastic Domains

**Figure 6:**
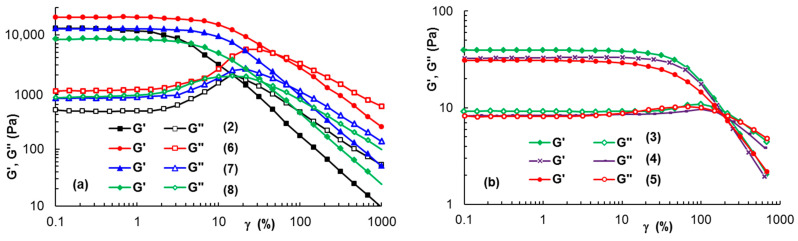
The viscoelastic moduli during amplitude sweep tests for (**a**) PL-containing gels; (**b**) XG solutions (10 rad/s, 37 °C).

Amplitude sweep tests were carried out to determine the upper strain amplitude values (γ_L_) limiting the linear domain of viscoelasticity and the corresponding shear stress values (Table 1). In the linear range of viscoelasticity, G′ and G″ were independent of the applied stain, as shown in Figure 6 for samples 2, 6, 7, and 8 (ω = 10 rad/s). For the PL-based hydrogels, a linear range of viscoelasticity was reached from very low strain values (γ below 0.1%) up to a value of strain above 1%. It can be observed that the addition of 1% XG (Figure 6a) or other polysaccharides (Table 1) determined an increase of γ_L_, i.e., the occurrence of an extended linear viscoelastic domain. Long XG chains in aqueous or salted solutions are in an entangled state and they are able to develop a greater resistance when increased strains are applied (Figure 6b), as compared with PL-based hydrogels with a micellar structure (Figure 6a).

The data obtained in the amplitude sweep tests also gave access to the yield stress, when the flow of the sample started [59]. Thus, σ_o_ can be considered as the upper value of shear stress, corresponding to the point that delimitates the linear and non-linear ranges of viscoelasticity. Usually, the value determined by this method overestimates the yield stress [54,56,60]. Thus, the σ_o_ values determined from these tests for XG solutions and PL-based hydrogels (Table 1) were higher compared with those determined in the continuous shear tests.

Samples withstanding higher deformations before yielding are considered more stretchable [56], thus the γ_L_ value can be correlated with the elasticity of materials. For a stress value below σ_o_, the material only underwent reversible (elastic) strain, without viscous (permanent) deformation. Above σ_o_, irreversible deformation of material occurs. From this point of view, XG addition to PL hydrogels seemed to be a suitable choice.

### 2.4. Self-Healing Behavior of Pluronic® F127 Hydrogels in the Presence of XG Chains

An important requirement of injectable hydrogels is self-healing ability; a fast recovering of the non-deformed state once they are injected in the damaged site, avoiding a sudden release of drugs or encapsulated cells after injection [24,61,62]. Similarly, it is necessary for 3D printed materials to possess high thixotropy, i.e., the viscosity (or other rheological parameters) quickly decreases when shear forces are applied, and the initial value is recovered very quickly when the shear forces are removed. From a structural point of view, the intermolecular interactions that ensure network formation recover rapidly to a high degree after printing [56]. When a hydrogel is submitted to high shear forces in a syringe needle, the network structure is strongly disturbed and the sample behaves as a fluid-like material. However, with the cessation of the external forces, the structural integrity must be recovered in order to maintain the functionality of biomaterials. Moreover, the shear forces applied to gels during their use affect the cell viability [54,63,64,65]. In such cases, soft materials with yield stress, shear thinning, and self-healing behavior are suitable materials.

**Figure 7:**
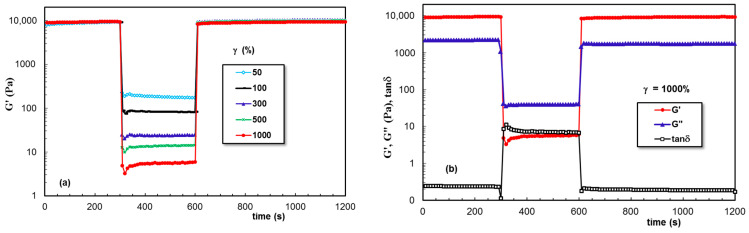
Illustration of thixotropic behavior for sample 2 at 37 °C: (**a**) Elastic modulus as a function of time during successive step strain measurements at low (1%), high (50%, 100%, 300%, 500%, 1000%), and again low (1%) values of γ; (**b**) G′, G″, and tanδ for γ = 1000%.

Figure 7 gives the data obtained for PL hydrogel (sample 2) in the oscillatory three-step strains experiments (for ω = 10 rad/s), when the strain values were successively switched each 300 s from a low amplitude (γ = 1%) to a high amplitude value and again to a low γ value of 1%. The viscoelastic parameters (G′, G″, and tanδ) were monitored as a function of time. High values of applied strain during the second step were chosen in the nonlinear viscoelastic regime (according to Figure 6): 50%, 100%, 300%, 500%, and 1000%, and in these conditions, a liquid-like behavior was registered (G′ became lower that G″ and tanδ > 1). Sample 2 showed nearly complete recovery after applying high deformations, and the time required for structure recovery was very short (on the order of a few seconds).

**Figure 8:**
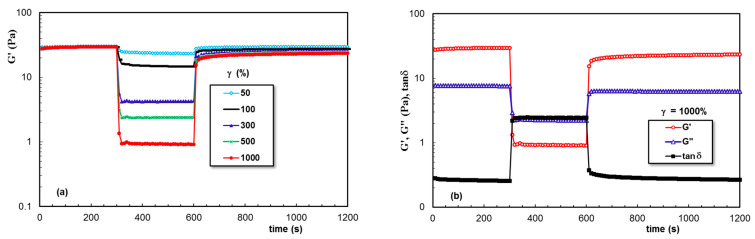
(**a**,**b**) As Figure 7, but for sample 3.

Figure 8 presents the behavior of the entangled XG aqueous solution (sample 3). The strong intermolecular interactions determined a solid-like behavior at a low γ value (1%). They were perturbed by the high deformations during step 2 and partially recovered when the external forces were removed. A similar behavior was observed for samples 4 and 5, but the recovery during step 3 was smaller compared with that of sample 3.

**Figure 9:**
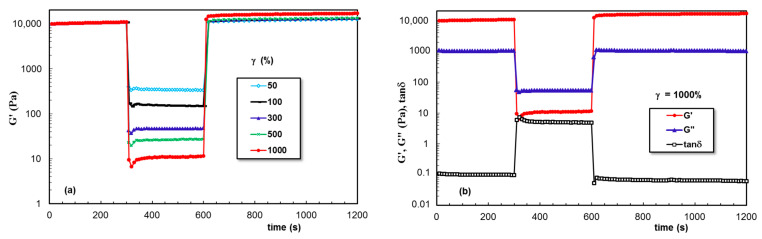
(**a**,**b**) As Figure 7, but for sample 6.

**Figure 10:**
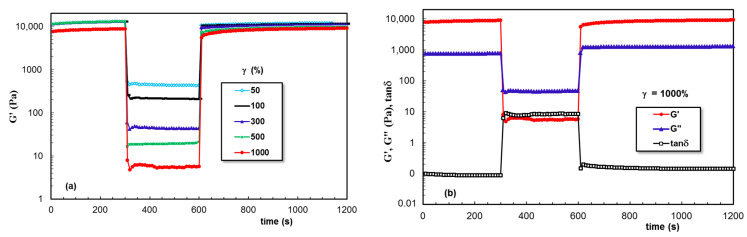
(**a**,**b**) As Figure 7, but for sample 7.

**Figure 11:**
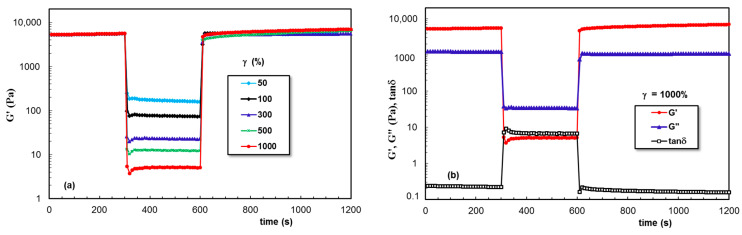
(**a**,**b**) As Figure 7, but for sample 8.

The data obtained for PL hydrogels in the presence of XG dissolved in aqueous, NaCl, and PBS solutions are given in Figure 9, Figure 10 and Figure 11, respectively. A remarkable behavior was depicted for sample 6, for which the network strength increased after each deformation cycle. This feature was not found in the presence of NaCl, when the network became weaker after increased deformations were applied. However, a good stability and total recovery of rheological parameters was observed for sample 8.

A possible explanation for these results could be the ability of XG chains to penetrate into the PL micellar network and to improve both the viscous and the elastic response, even in conditions of high applied strains. The XG concentration considerably influenced its viscosity and thermal history in aqueous solutions [66]. The addition of different amounts of XG to PL aqueous solutions (samples 6, 9–11) influenced the gelation temperature and rheological characteristics of the hydrogels (Table 1). It was observed that the addition of 1% XG induced improved rheological characteristics for PL/XG hydrogels compared to the neat PL sample.

### 2.5. Self-Healing Behavior of PL Hydrogels in the Presence of Other Polysaccharides

**Figure 12:**
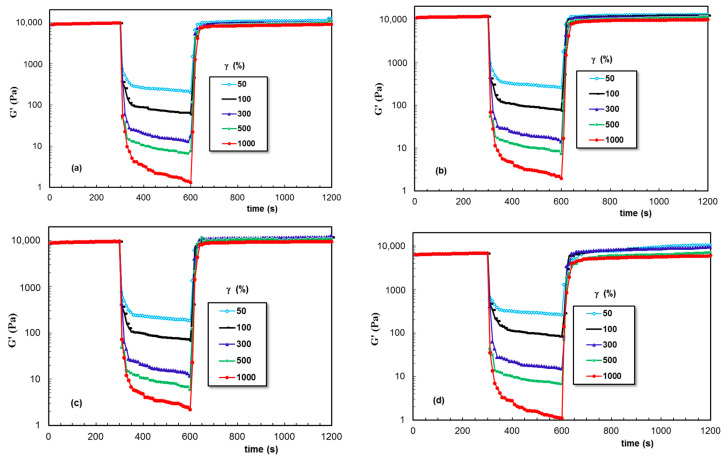
As Figure 7, but for samples (**a**) 12; (**b**) 13; (**c**) 14; (**d**) 15.

Due to the unusual thixotropic behavior observed for XG, the self-healing behavior was analyzed for PL gels containing other polysaccharides: Alg, κ-Carr, GG, and CS (Figure 12). For all these hydrogels (samples 12 to 15, Table 1), the high deformations applied during step 2 determined a more pronounced decrease in the viscoelastic parameters, suggesting that the network strength was smaller compared to the PL/XG hydrogels (Figure 9, Figure 10 and Figure 11).

In order to compare the effect of each polysaccharide addition, we defined the self-healing efficiency as

(1) $$\mathrm{SH}\;(\%)=\frac{G\prime_{\gamma }}{G\prime_{\mathrm{rest}}}\;\;\times \;\;100$$

where G′_γ_ is the elastic modulus registered after applying a given value of γ, and G′_rest_ is the initial value of G′ obtained at low γ value, in the linear range of viscoelasticity.

**Figure 13:**
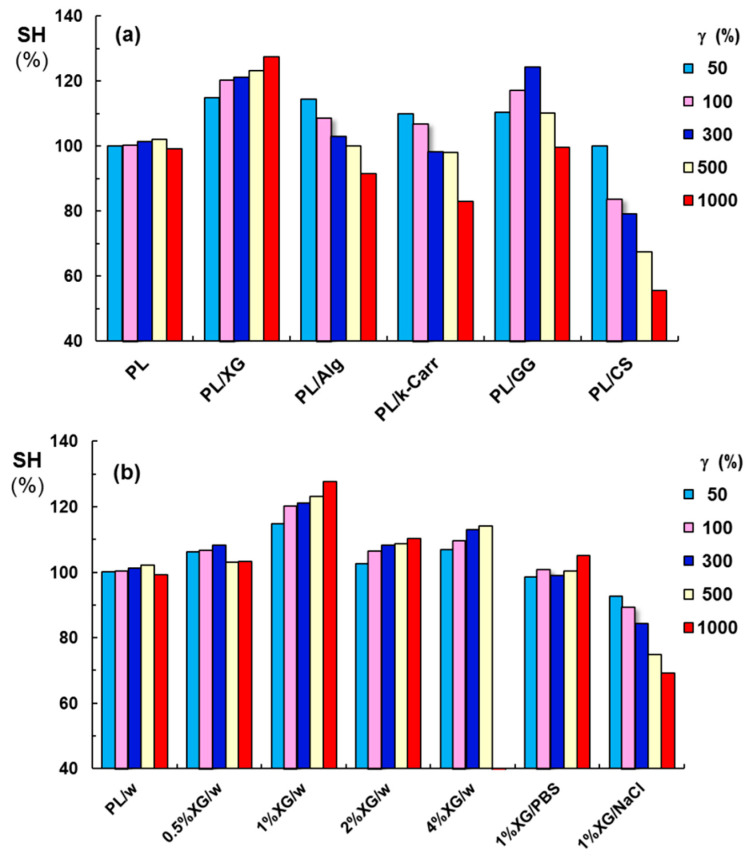
Illustration of the influence of polysaccharide addition on the self-healing efficiency of PL hydrogels after applying three step cycles of various levels of strain during step 2 (γ = 1% for the steps 1 and 3): (**a**) PL and PL/polysaccharides in water and (**b**) PL/XG hydrogels obtained by adding various XG concentrations in water (w) or 1% XG in salted solutions (0.1 M NaCl or PBS).

Figure 13 presents the SH parameter for PL-based gels in the absence and in the presence of various polysaccharides, in conditions of various strain values applied during step 2. According to the thixotropic behavior investigated in similar conditions for all PL-containing samples, the pure PL hydrogels (in the absence of polysaccharides) presented a complete and rapid structure recovery. Besides XG addition, GG included into PL hydrogels determined an increase in PL network strength, but only for γ ≤ 300%; for γ values of 500% or 1000%, the G′ values decreased, but they were still above the initial value registered for γ = 1%. Alg or κ-Carr addition was efficient for low values of strain 50% or 100%, but the structure was disturbed at high values of strain (1000%). Similar effect was recently reported for PL samples in presence of carboxymethyl pullulan for concentrations below 1% [67]. PL gels in the presence of 1% CS or XG in NaCl solution presented modest self-healing ability, the structure was strongly perturbed by applying high strains that belonged to non-linear range of viscoelasticity, and the network strength decreased after each cycle of deformation.

**Figure 14:**
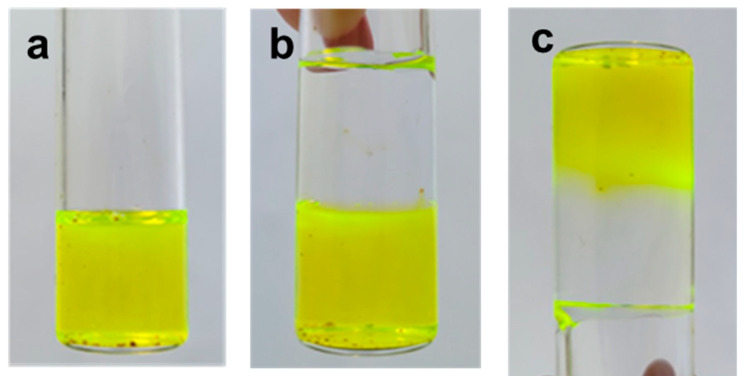
Macroscopic illustration of the self-healing behavior for sample 6: (**a**) sample 6 colored with fluorescein; (**b**) two pieces of hydrogel after they were put into contact; and (**c**) the aspect of the self-healed hydrogel after 12 h.

The addition of different XG amounts to PL samples revealed the existence of an optimum XG concentration (Figure 13b): thus, sample 6 (1% XG added to PL gels) presented a considerably improved gel strength (Table 1), which increased after applying high strains (the values of elastic modulus increased when increasing the applied strain). The macroscopic self-healing of this sample is illustrated in Figure 14. The PL/XG hydrogel was examined by taking pictures of two pieces of hydrogel, one of them colored with fluorescein (Figure 14a), at different times after putting them in contact (Figure 14b,c). This behavior was attributed to the re-formation of the hydrophobic interactions and hydrogen bonds through the spontaneous diffusion of PL micelles and slow rearrangements of long XG chains surrounded by PL micelles at the site of the applied strain.

The composite PL/XG gels were transparent and stable over time at room temperature. The addition of natural gums to the PL-based hydrogel influenced the gelation and the drug release profile of PL-based gels [38]. Thus, the main potential application of these hydrogels is their use as drug-delivery vehicles for ophthalmic [37,38], buccal [39,57], or transdermal drug delivery applications [40].

Another aspect concerns the influence of environmental conditions on the gel properties [49,50,51]. The solvent can change the mechanical performance of self-assembling hydrogels, as it was reported for triazole-linked lipid derivatives, suitable for topical formulations [68].

## 3. Conclusions

Hydrogels containing Pluronic® F127 in the presence various polysaccharides were prepared and investigated through rheological measurements. In situ gelation of PL hydrogels in the presence of XG was followed in detail. The PL/XG hydrogels were shear thinning, and presented yield stress and improved self-healing ability, important characteristics for injectable materials or bioinks. An optimum concentration of 1% XG was depicted in the rheological investigations. Salt addition determined the formation of aggregates and decreased the PL-based performances.

The excellent self-healing ability induced by XG chains was not observed when this biomacromolecule was replaced with other polysaccharides. The influence of alginate, κ-carrageenan, gellan, or chitosan addition was investigated in similar conditions. The data analysis suggested that the network structure and rheological properties of the PL micellar hydrogel can be tuned by selecting an appropriate polysaccharide structure. Thus, good results were obtained in the presence of gellan gum, alginate, and κ-carrageenan, but the ability to recover the initial structure decreased after applying high strains. A modest viscoelastic behavior and self-healing ability were observed when chitosan was added to PL gels and this may have been due to the presence of acetic acid in the system.

In conclusion, the present study was focused on obtaining homogeneous systems in sol state and on the investigation of the sol–gel transition and hydrogel properties, with an emphasis on viscoelastic characteristics and self-healing behavior. The current findings could provide a good starting point for discussion and further research.

## 4. Materials and Methods

### 4.1. Materials

All polymer samples, Pluronic® F127 (denoted PL), and polysaccharides: xanthan gum (XG), sodium alginate (Alg), kappa-carrageenan (κ-Carr), gellan gum (GG), and chitosan of low viscosity (CS) were purchased from Sigma-Aldrich Co. (Taufkirchen, Munich, Germany).

**Figure 15:**
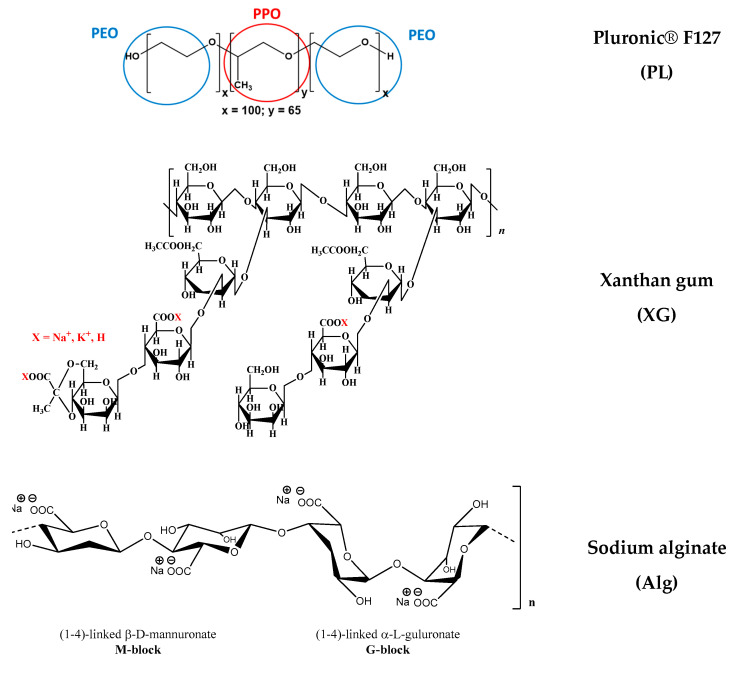

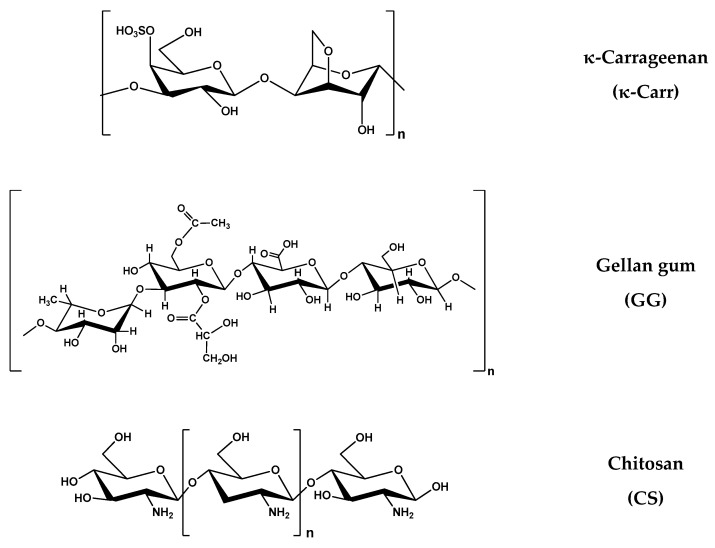
The chemical structure of the polymers.

PL is a triblock copolymer with a central hydrophobic poly(propylene oxide) (PPO) block and two hydrophylic poly(ethylene oxide) (PEO) blocks, i.e., (PEO)x-b-(PPO)y-b-(PEO)x, with x = 100, y = 65. The XG sample had a high molecular weight: M = 2 × 10^6^ g/mol. Three polysaccharides presented closed values of molecular weight: GG: M = 5 × 10^5^ g/mol, κ-Carr: M = 4.85 × 10^5^ g/mol and Alg: M = 4.70 × 10^5^ g/mol. The CS sample had a M of 2 × 10^5^ g/mol. The chemical structures of the polymers used in hydrogel preparation are given in Figure 15.

### 4.2. Sample Preparation

PL and polysaccharides (excepting CS) were dissolved in Millipore water. Other XG samples were prepared in 0.1 M NaCl and PBS solution. CS was dissolved in 1% acetic acid solution. Stock solutions of PL (20%, wt. and 16.83%) and polysaccharides (1%) were prepared by mixing the polymer with the solvent. For XG, samples of various concentrations (0.5%, 1%, 2%, and 4%) were prepared in aqueous solutions.

PL solution was prepared at low temperature and then stored at 4 °C. Polysaccharide solutions were freshly prepared at room temperature using a weak magnetic stirring system and stored at 4 °C for 24 h, excepting the GG sample, which was kept at room temperature. The biomolecules were added to homogeneous 20% PL solutions and the total concentration of polymer (PL and polysaccharide) in all samples was 16.83% wt.

### 4.3. Rheological Investigation

Rheological measurements were carried out with a MCR 302 Anton-Paar rheometer (Graz, Austria) using a plane–plane geometry (the upper plate of 50 mm, gap of 500 μm) and Peltier device for temperature control.

The sol–gel transition was investigated using solutions stored at 5 °C and then introduced into the geometry of the rheometer, which had previously been thermostated at 5 °C. Temperature seep tests were carried out at a heating rate of 1 °C/min for an oscillation frequency (ω) of 10 rad/s and strain amplitude (γ) of 1%. The elastic (G′) and viscous (G″) moduli were determined, and they give information about the stored and dissipated energy during one cycle of deformation, respectively. A useful parameter expressing the degree of viscoelasticity is the loss tangent (tanδ) determined as the G″/G′ ratio.

During the gelation experiments, the temperature was first set at 5 °C for 120 s and then suddenly switched to 37 °C. The viscoelastic parameters G′, G″, and tanδ were monitored as a function of time at a constant oscillation frequency (ω) of 10 rad/s and strain amplitude (γ) of 1%. The time-dependent self-assembly process was only observed with addition of chitosan/acetic acid solution and XG/0.1 M NaCl. For these systems, incubation at 37 °C for 24 h was required. For the other systems, the self-assembling was relatively fast, an incubation at 37 °C for 1 h was sufficient to reach equilibrium.

The gels thermostated at 37 °C were investigated in amplitude sweep tests carried out to determine the upper limit of strain, γ_L_, for the linear viscoelastic regime and the yield stress value (σ_o_). The shear viscosity was determined in stationary shear conditions for shear rates ($\dot{\gamma }$) varying from 0.01 s^−1^ to 1000 s^−1^.

Self-healing tests were carried out in strain step oscillatory mode for ω = 10 rad/s and the step strains varied every 300 s from low (1%) to high values (50%, 100%, 300%, 500%, and 1000%), in the nonlinear range of viscoelasticity and again the low step of strain (1%).

### 4.4. Morphology of Hydrogels

The PL, XG, and composite PL/XG samples were frozen at −55 °C and dried using a freeze drier (BenchTop Pro with Omnitronics ^TM^, SP Scientific) for 48 h. Dry samples were coated with a 6 nm platinum layer using a Leica EM ACE200 Sputter coater prior to microscopic examination to improve the electrical conductivity and prevent charge buildup. The morphology was analyzed using a Verios G4 UC Scanning Electron Microscope (Thermo Scientific, Brno, Czech Republic), operating at 10 kV in high-vacuum mode with a backscatter electron detector, ABS (Angular Backscattered Detector). The SEM images were observed at various magnifications.

## Figures and Tables

**Table 1:** Sample composition, gelation temperature (T_gel_), and the values of rheological parameters determined in different shear conditions at 37 °C.

Sample Code	Polysaccharide Solution of 1% (wt.)	PL Concentrationin Water (% wt.)	T_gel_^(a)^	G′ ^(b)^ (Pa)	G″ ^(b)^ (Pa)	tanδ ^(b)^	σ_o_^(c)^ (Pa)	σ_o_^(d)^ (Pa)	γ_L_^(d)^ (%)
1	-	20	21.6	17,200	4200	0.2442	197.5	220	1.49
2	-	16.83	26.9	10,923	2780	0.2545	189	209	1.55
3	1% XG/water	-		20.9	7.03	0.3364	4.41	12.3	25.3
4	1% XG/0.1 M NaCl	-	-	32.52	8.47	0.2604	2.09	11.5	21.7
5	1% XG/PBS	-	-	56.4	13.1	0.2323	1.72	2.99	10.1
6	1% XG/water	20	25.4	13,100	763	0.0582	1080.4	1010	6.77
7	1% XG/0.1 M NaCl	20	23.6	10,800	1057	0.0979	273.1	338	4.58
8	1% XG/PBS	20	29.1	6643	1322	0.1990	104.9	133	1.38
9	0.5% XG/water	20	24.8	9710	832	0.0857	173	152	2.15
10	2% XG/water	20	28.0	9101	914	0.1004	159	180	2.17
11	4% XG/water	20	29.0	7864	793.4	0.1009	147	169	2.17
12	Alg/water	20	23.4	8990 ^(e)^	2450 ^(e)^	0.2725	306.4	422	2.74
13	κ-Carr/water	20	23.5	10,300 ^(e)^	2990 ^(e)^	0.2903	204.48	217	1.73
14	GG/water	20	23.1	8680 ^(e)^	2310 ^(e)^	0.2661	277	311	2.51
15	CS/1% acetic acid solution	20	24.3	8800 ^(e)^	3300 ^(e)^	0.3750	108	124	1.73

^(a)^ temperature sweep test, heating rate of 1 °C/min; ^(b)^ frequency sweep test, γ = 1% and ω = 1 rad/s; ^(c)^ continuous shear flow (shear stress imposed); ^(d)^ amplitude sweep test, ω = 10 rad/s; ^(e)^ previous data [18].

## Data Availability

Data are available on request.
